# Exocentric and Egocentric Views for Biomedical Data Analytics in Virtual Environments—A Usability Study

**DOI:** 10.3390/jimaging10010003

**Published:** 2023-12-23

**Authors:** Jing Ng, David Arness, Ashlee Gronowski, Zhonglin Qu, Chng Wei Lau, Daniel Catchpoole, Quang Vinh Nguyen

**Affiliations:** 1 School of Psychology, Western Sydney University, Penrith, NSW 2750, Australia; 19777001@student.westernsydney.edu.au (J.N.); d.arness@westernsydney.edu.au (D.A.); 19316534@student.westernsydney.edu.au (A.G.); 2 School of Computer, Data and Mathematical Sciences, Western Sydney University, Penrith, NSW 2751, Australia; 18885806@student.westernsydney.edu.au (Z.Q.); 18687330@student.westernsydney.edu.au (C.W.L.); 3 Tumour Bank, Children’s Cancer Research Unit, Kids Research, The Children’s Hospital at Westmead, Westmead, NSW 2145, Australia; daniel.catchpoole@health.nsw.gov.au; 4 School of Computer Science, Faculty of Engineering and IT, The University of Technology Sydney, Ultimo, NSW 2007, Australia; 5 School of Computer, Data and Mathematical Sciences and MARCS Institute, Western Sydney University, Penrith, NSW 2751, Australia

**Keywords:** virtual reality, virtual environment, exocentric visualization, egocentric visualization, biomedical data

## Abstract

Biomedical datasets are usually large and complex, containing biological information about a disease. Computational analytics and the interactive visualisation of such data are essential decision-making tools for disease diagnosis and treatment. Oncology data models were observed in a virtual reality environment to analyse gene expression and clinical data from a cohort of cancer patients. The technology enables a new way to view information from the outside in (exocentric view) and the inside out (egocentric view), which is otherwise not possible on ordinary displays. This paper presents a usability study on the exocentric and egocentric views of biomedical data visualisation in virtual reality and their impact on usability on human behaviour and perception. Our study revealed that the performance time was faster in the exocentric view than in the egocentric view. The exocentric view also received higher ease-of-use scores than the egocentric view. However, the influence of usability on time performance was only evident in the egocentric view. The findings of this study could be used to guide future development and refinement of visualisation tools in virtual reality.

## 1. Introduction

Data visualisation is the most effective method for explaining and conveying complex biomedical data [1]. The rapid growth of biomedical data in complexity and volume creates new challenges in effectively and accurately presenting data [2]. Data visualisation can assist users in leveraging cognitive strengths, such as pattern recognition, while overcoming cognitive limitations, including remembering and calculating strings of numbers.

Virtual Reality (VR) is gaining popularity in the gaming industry due to the availability of new and cheaper head-mounted devices that offer greater levels of immersion as well as improved graphics and sound quality [3]. VR users can immerse themselves in the data, benefit from the larger space, fewer distractions, more significant natural interactions, and instinctively analyse multidimensional data. Virtual environments, by nature, provide interaction and multivariate data can be viewed differently because users can naturally alter the visualisation’s perspective based on their viewpoint. Interacting with data in a virtual environment can be much more natural because users can grasp and pull the visualisation closer for a more in-depth look with fewer distractions, which is crucial for medical education [4].

An immersive dashboard displays multivariate data through an interactive interface composed of coordinated views to assist users in analysing, monitoring, making decisions, and communicating data [5]. Due to the precise, low-latency motion tracking, users can move around the data, switch from an overview level to the details, and step inside the visualisation [6]. Thus, VR enables users to analyse the complexity of data holistically, which was previously thought impossible with traditional data visualisation methods. This improves comprehension, decision-making, and task completion in clinical and educational settings [7].

Differently from conventional screens, VR environments unleash the capability further to allow us to see and explore the data differently and immersively from the inside out [8]. This can also show hidden information in the mass cloud which would not be possible in a traditional way. The inclusion of aural, haptic, and kinaesthetic in VR environments can also benefit the analytics because the multisensory inputs are naturally encoded within human beings, and when used correctly. Thus, immersive visualisation can improve the perception of information and cognitive processing in comparison with 2D displays. It is useful to test out to see if existing theories and analytics work better in such an immersive environment to unleash its full potential.

In the VR environment, the user can view and interact with the information in the space using two frames of reference or exocentric and egocentric views. The exocentric view represents external spatial relations independent of the observer’s position [9] which users can shift to a bird’s-eye view above and behind them. The view allows the user to gain better awareness on their location within the environment which is useful for the interaction and engagement. In contrast, the egocentric view relates to the representation of orientation and location to the observer’s perspective, such as the head, eye, or body coordinates [10]. In order words, egocentric view provides a view of an object or space standing from within the visualisation. Given the possibility of spatially manipulating data, frames of reference such as exocentric view and egocentric view have different strengths for learning in a VR environment study [11]. The use of exocentric and egocentric views can influence how visualisations can be effectively perceived by the users.

Despite the need for innovative ways to visualise health data to fully comprehend vast amounts of health information, there have been limited evaluation studies on the effectiveness of genomic data visualisation methods and data presentation strategies, especially in a VR environment. To the extent of the authors’ knowledge, no existing usability study has been carried out to evaluate the effectiveness of two frames of reference for health data in the VR environment. The present study bridges the gap by gaining a better understanding of the exocentric and egocentric views of genomics and biomedical data on VR and identifying potential design improvements in immersive analytics.

The paper explicitly evaluates the efficiency and functionality of exocentric and egocentric views as VR features, as well as their impact on time performance via usability in a virtual environment. The time performance was measured using the visual analytics task in a VR environment, an approach applied to health data analysis that employs interactive visualisation and human perception to make sense of large amounts of information to measure user performance and experience. The user performance and experience were measured using health data analytics in a VR environment using the VROOM tool [8], which was designed to evaluate cancer patient data. The following hypotheses were tested in this study:

- H1. The exocentric view of health data will result in faster time performance in the visual analytics task compared to the egocentric view.
- H2. The exocentric view of health data will have higher ease-of-use scores compared to the egocentric view.
- H3. The usability of the exocentric and egocentric views will be positively related to the time performance of the visual analytics task for each respective view.

The rationale for Hypothesis 1 (H1) was derived from Yang et al.’s study [12], showing that the exocentric view enhances faster time performance due to its ability to present health data without distortion. The study in [13] revealed that the egocentric frame of reference was more effective in increasing task performance than the exocentric frame of reference for storing virtual objects in a virtual environment. Participants could better remember and associate the information with virtual objects by referencing it with parts of their bodies (i.e., egocentric) than parts of the objects on the table or environment (i.e., exocentric), which motivate the study for Hypothesis 2 (H2). Our study also aligns with the Self-Determination Theory [14] to identify the relatedness of performance and analytical tasks (Hypothesis 3 (H3)). Poor task design can lead to low performance and user experience.

## 2. Related Work

The vast amount of information generated from patient genetic profiles have made interactive visualisation crucial to allow us to view, explore, and make sense of the data. Genomics data analytics and visualisation have been developed on traditional displays, such as in [15,16], mobile devices, such as in [17], and large and high-resolution immersive environments [18]. Unfortunately, there are still limited research works on using VR for interaction with biomedical data about patients and in clinical decision making. VR has been mainly used in the modelling of Computed Tomography (CT) and Magnetic Resonance Imaging (MRI) data (such as in [19,20]), patient care and pain treatment (such as [21,22]), and those in the summary in the recent reviews [23].

Adopted works in biomedical data analytics in VR environments include CellexalVR [24], StarMap [12], MinOmics [25], and BioVR [26]. CellexalVR was designed to deliver a visualisation of single-cell RNA sequencing (RNASeq) data in the immersive environment with multiple statistical data analytics toolsets to take advantage of the unlimited space in the virtual environment. StarMap also provides an immersive visualisation for single-cell RNASeq data, but it focuses on the mobile web browser on a VR-enabled smartphone. MinOmics visualises proteomic and transcriptomic data using UnityMol platform to manage the biological data from storage to analysis. BioVR extends UnityMol for visualising DNA and RNA sequencing data, multi-omics data, and protein structures in virtual reality. However, the above methods do not provide users with a visualisation to deep-dive into the genomic similarity with the patient treatment history required for the clinical assessment which are crucial for analysis in the whole cohort.

**Figure 1:**
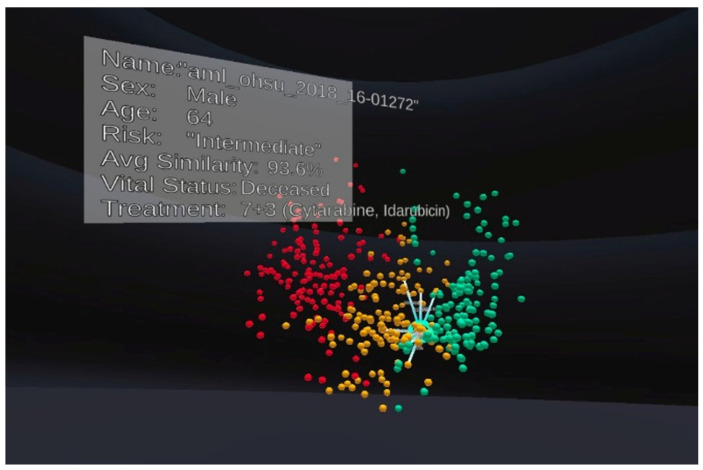
An example of the exocentric view in our experiment. Each patient is represented as a spherical object. Colours are used to indicate the risk level, including high-risk (red), medium-risk (yellow) and low-risk (green).

**Figure 2:**
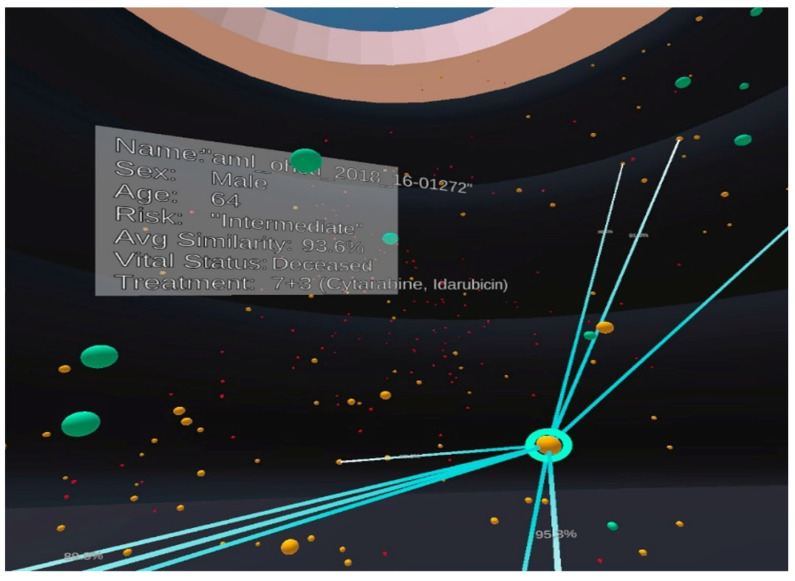
An example of the egocentric view in our experiment with the same patient population and visual mapping as shown in Figure 1. Colours are used to indicate the risk level, including high-risk (red), medium-risk (yellow) and low-risk (green).

**Figure 3:**
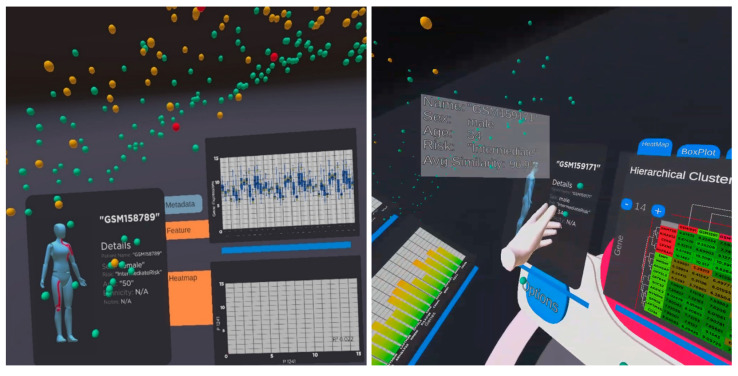
An example of analytical views on selected patients to view patients’ biomedical and genomics information. The left figure shows a female patient with a box plot and a scatter plot. The right figure shows another male patient and the heatmaps and bar chart of the significant genes for detailed analysis.

**Figure 4:**
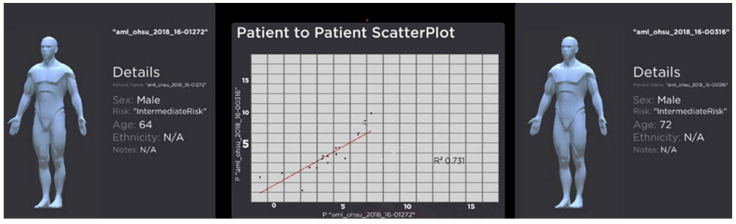
An example of an interaction when two patients are selected for analysis. The view shows the metadata for the patient, such as age, gender, and ethnicity. The scatterplot shows the similarity between the selected patients regarding their gene expression. R-square is the squared multiple correlation or coefficient of determination of the linear regression model indicates. A higher R-square value (closer to 1) means the better the linear regression or a higher similarity between the patients.

The recent work VROOM [8] employs multiple state-of-the-art methods in computational analytics, 3D visualisation, immersive design principles, and visual analytics with intelligent decision support to provide a complete and comprehensive tool for analysing cancer patient cohorts in virtual environments. The tool also employs traditional 2D charts (such as scatter plots, descriptive statistical information, linear regression, box plot, and heatmap) to provide familiar and meaningful views to the domain users (see examples in Figure 1, Figure 2, Figure 3 and Figure 4).

Despite the need for innovative ways to visualise biomedical data to comprehend vast amounts of health information fully, the studies in egocentric and exocentric views have not yet been applied in a medical domain. It is useful to understand the effect of the two designs for visualisation and exploration that can potentially guide the development of future visualisation tools in VR.

Cognitive psychology literature has shown that a user can associate objects with information to better remember the object’s position, features, and orientation in the space through the two frames of reference or views: exocentric and egocentric [13]. The exocentric reference frame represents external spatial relations independent of the observer’s position. In contrast, the egocentric reference frame relates to the representation of orientation and location to the observer’s perspective, such as the head, eye, or body coordinates [10]. Khadka and Banic’s study examined how a method of virtual object storage (exocentric versus egocentric) affects memory [13]. Their results revealed that the egocentric reference frame was more effective in increasing task performance than the exocentric reference frame for storing virtual objects in a virtual environment. Participants were able to better remember and associate the information with virtual objects by referencing it with parts of their body (i.e., egocentric) than parts of the objects on the table or environment (i.e., exocentric). These findings indicate that users can associate objects with information to better remember their features, location, and attributes in relation to their bodies during cognitive tasks.

The exocentric and egocentric views have distinct characteristics for learning in a VR environment due to their ability to manipulate data spatially [11,12,27]. Yang et al. investigated the exocentric and egocentric views in the context of virtual maps and globes used in geographic visualisations [12]. Their research examined the effectiveness of two interactive views, such as exocentric and egocentric globes, on three geospatial analysis tasks involving area and distance comparisons, as well as the estimation of orientation between two locations in terms of time and accuracy. VR users can immerse themselves in the data, benefit from the larger space, fewer distractions, more significant natural interactions, and analyse multidimensional data instinctively. In alignment with a recent study [28], Yang’s findings also showed that the exocentric globe was the best overall choice for most tasks because it could present geospatial data without distortion. The egocentric globe, on the other hand, was the least effective in almost all tasks. While it featured the most immersive visualisation, it also had the most significant perception distortion, which required more effort in body interactions, such as turning one’s head more frequently. Differently from the above, this study evaluates the efficiency and functionality of exocentric and egocentric views focusing on genomics and biomedical data, where we also provide greater analytical interaction and visual analytics components in the two views.

## 3. Method

The present study employed a quantitative design to evaluate usability and user performance on exocentric and egocentric views for analysing and exploring genomic data in a VR environment. To assess participants’ performance, quantitative methods such as dependent sample *t*-tests and multiple regression were used to investigate the usability of 3D visualisation methods that contribute to time performance and user preference for visual analytics tasks. A short post-study questionnaire was used at the end of the experiment to evaluate the usability and preferences for 3D visualisation methods, such as exocentric and egocentric views, on health data.

The VROOM tool on Oculus Quest [8] provides an interactive, immersive visualisation of gene expression for cancer patients from https://www.cancer.gov/ccg/research/genome-sequencing (accessed on 10 November 2023). Each patient’s cancer severity is represented by a spherical object, with green representing low risk, orange representing medium risk, and red representing high risk. The tool allows the user to select individual patients and their genomic features to perform a patient-to-patient comparison based on bioinformatics, gene data, and treatment. Most importantly, participants were instructed to complete the tasks using the two frames of reference (i.e., exocentric or egocentric) assigned to them at the time. Figure 1 and Figure 2 show examples of screenshots of the VROOM’s exocentric and egocentric views, respectively. The exocentric frame of reference in Figure 1 allows users to view the entire data cloud (patients’ information our study) in one go compactly in the front-of-eye view. The egocentric frame of reference in Figure 2 requires users to focus on portions of the data cloud of specific interest in a more immersive manner where some information can be hidden at the current eyes’ viewport.

### 3.1. Participants

This study included 38 individuals (21 females, 15 males, 2 non-binary) who were primarily tertiary students. Participants were primarily first-year psychology students identified through the university’s SONA research participation system. All participants were between 18 and 71 (M = 30.29, SD = 16.16), had a normal or corrected vision, and had no existing or underlying medical conditions that interfered with their ability to wear a VR headset and use hand controllers. Furthermore, 45% of participants reported no prior experience with VR technology, 42% reported little use, 8% reported occasional use, and 5% reported frequent use. Participants were incentivised with course credit or remunerated with an AUD$30 voucher.

### 3.2. Measures and Materials

Before the study, participants completed an online Qualtrics survey with demographic questions about their gender, highest level of education, and general experience with the VR head-mounted display (HMD).

#### 3.2.1. Visual Analytics Tasks

The visual analytics experiment consisted of five tasks and two questions involving the interaction and selection of visual items in the VR space as shown in Table 1. The designed tasks aimed to assess user perception and user interaction with specific objects (i.e., patients) in the cohort in egocentric and exocentric views. The questions were used to evaluate user readability of analytical charts in the two views. The tasks and questions are designed with simplicity so that the participants from various disciplines can follow the experiment and perceive the information.

Participants were timed based on how long it took to complete each task and answer the associated question(s), which were then added up together to calculate the total time. The participants’ accuracy was determined by their ability to answer the questions correctly. Figure 3 and Figure 4 depict examples of a visual analytics view during the analytical experiments. Figure 3 shows the entire patient population in the space and the analytical panels of a selected patient with genomics and biomedical information. The visual analytics uses a variety of common 2D charts in biology, including heatmaps, box plot, bar charts, and scatterplots on the immersive 3D space. This design strategy ensures domain familiarisation and is easy-to-use in the immersive visualisation. Figure 4 shows another view when two patients are selected for the comparison and analysis. The figure indicates the similarity in the patients’ genomics information and background information, as shown in scatterplots and details panels. A technical description of the visualisation, interaction and visual analytics components was presented in [8].

#### 3.2.2. Usability Measure

The self-report questionnaire assessed the perceived ease of use variable and measured the usability construct [10]. The questionnaire was scored on a 5-point Likert scale ranging from 1 (strongly disagree) to 5 (strongly agree). The questionnaire items were modified to reflect the current study’s context and the conditions under which the participants were required to complete their tasks. For example, an original item from the perceived ease of use measure “Learning to operate CHART-MASTER was easy for me” [29] was modified to “Learning to interact with the exocentric 3D-web-based VR simulation was easy for me” for the exocentric condition and “Learning to interact with the egocentric 3D-web-based VR simulation was easy for me” for the egocentric condition. All participants were instructed to complete the tasks and questionnaire.

#### 3.2.3. Preference Measure

The post-study questionnaire included four questions about identifying similarities and differences in users’ experiences with the VR tool, as well as egocentric and exocentric views. The questions included “Did you experience any discomfort while using the Oculus Quest Virtual Reality Headset?”, “Do you prefer the exocentric view or the egocentric view when analysing and exploring genomic data in VR?”, “Which of the visual design features support your usability?” and “Which visual design features stood out to you and were they helpful or distracting?”.

At the end of each set of statements, there was also space for participants to provide general feedback and comments about their experiences with each of the frames of reference. Finally, participants were asked which visual design features (exocentric versus egocentric view) they found useful when performing patient-to-patient comparison tasks.

### 3.3. Procedure

The experiments on exocentric and egocentric were counterbalanced to limit order effects in the within-subjects design (i.e., learning effects or fatigue). All participants completed the similar conditions, either beginning with the exocentric view and progressing to the egocentric view or beginning with the egocentric view and progressing to the exocentric view, and follow with the post-study questionnaire. Individual trials were conducted in a quiet classroom with at least a 3 × 3 m boundary, minimal distractions, and controlled lighting. Participants’ informed consent was documented, and they could withdraw from the study at any time if the HMD caused discomfort. The experiment took approximately 90 min and included a set of demographic questions on Qualtrics, a short training on using VR, exocentric versus egocentric views on VR, and a post-study questionnaire on Qualtrics at the end of the experiment.

Prior to the first trial, all participants completed a comfort settings checklist with the HMD and a 15 min training phase to introduce them to the study’s 3D VR simulation. The training phase involved using the hand controllers, navigating the visualisation, and performing relevant functions such as selecting a patient of interest using the exocentric view. During this phase, participants were encouraged to ask questions and clarify their understanding of using the HMD and hand controllers. The testing phase began after participants felt comfortable using the hand controllers to complete the task in the data visualisation program.

In the testing phase, participants were required to complete three tasks based on a clinical scenario for each of the two views. Each task instruction was read aloud to the participants, who were encouraged to complete the given task promptly and accurately. The experimenter timed each task and reset the timer after each. Participants were assisted in removing the HMD from their heads after completing all tasks. Afterwards, participants were invited to complete a post-study questionnaire regarding their experiences and preferences for using each view to explore large data sets. The study was completed when the participant verbally confirmed that they had completed the post-study questionnaire.

## 4. Results

Two paired sample *t*-tests were used to compare time performance and participant preference when using the exocentric and egocentric views. Multiple regression was used to assess the usability of the exocentric and egocentric views in relation to time performance for each view. The data set was screened for data entry accuracy, missing data, outliers, and violations of parametric assumptions. Scores for items 1 and 3 in perceived ease of use were reverse-coded. The initial data screening revealed no missing data and appeared within plausible ranges. The paired sample *t*-test and multiple regression analysis assumptions were also tested. Data screenings with and without outliers were performed, and comparative testing confirmed that the presence or absence of outliers had no significant difference or impact on the results.

### 4.1. Time

A paired sample *t*-test was performed to determine whether time performance in the exocentric view would be faster than in the egocentric view. The initial paired sample *t*-test was conducted with outliers retained, and then the test was repeated with outliers modified to be one unit more extreme than the next most extreme score [30]. The two outliers were the times recorded in the egocentric view for participants 10 and 15.

**Figure 5:**
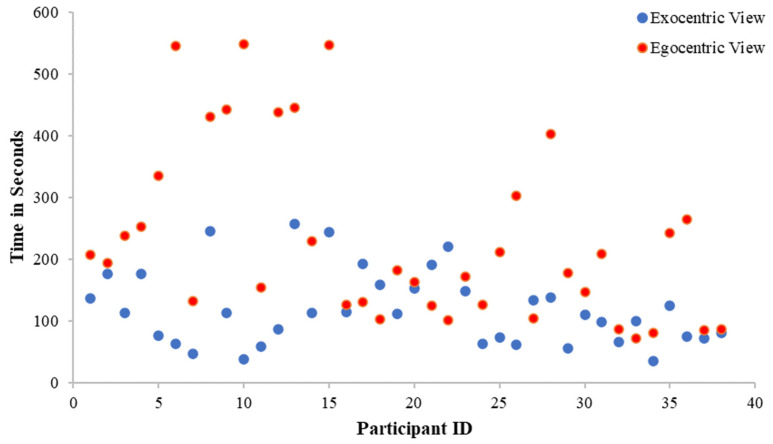
Time performance in exocentric and egocentric views. The figure shows the participants spend less time completing the tasks in the exocentric view in comparison with the egocentric view.

The assumption of normality was met and the result was statistically significant, *t*(37) = −4.81, *p* < 0.001 (see Table 2). This indicated that the average time taken to complete tasks in the exocentric view (*M =* 119.19, *SD =* 59.81) was significantly quicker than the average time taken to complete tasks in the egocentric view (*M =* 232.96, *SD =* 143.49). Data for time performance in exocentric and egocentric views are presented visually in Figure 5, indicating increased efficiency when using the exocentric view, which supports H1.

### 4.2. Ease-of-Use Scores

A paired sample *t*-test was conducted to examine whether the exocentric view had higher ease-of-use scores than the egocentric view. The initial paired sample *t*-test was conducted with outliers retained and then re-run with outliers modified to be one unit more extreme than the next most extreme score [30]. The two outliers were the ease-of-use scores recorded in the exocentric view for participants 6 and 9. The assumption of normality were met and the result was statistically significant, *t*(37) = 2.80, *p* = 0.008 (see Table 3). This indicated that the mean ease-of-use score for the exocentric view *(M* = 4.0, *SD* = 0.8) was higher than the mean ease-of-use score for the egocentric view *(M =* 3.6, *SD* = 1.0). Thus, H2 is supported.

### 4.3. Influence of Usability on Efficiency

With alpha set as 0.05, a multiple regression was performed between time performance as the criterion, and the two predictor variables entered as the average of the ease-of-use scores for the exocentric view and the average of the ease-of-use scores for the egocentric view. The results in Table 4 revealed that the overall model was significant *F*(2, 35) *=* 6.36, *p* = 0.004, with the average of the ease-of-use scores in the egocentric view explaining 26.7% of the variance in the time taken to complete each task, *R^2^* = 0.27.

However, as shown in Table 5, coefficients indicated that the averages of the ease-of-use scores were only significant independent predictors in the case of the egocentric view (*B* = −67.82, *p* = 0.01). In contrast, the exocentric view was not significant on task completion time (*B* = −10.95, *p* = 0.738). No evidence of multicollinearity between the exocentric usability average score and the egocentric usability average score was detected as Variance Inflation Factor (VIF) scores were below 10 (VIF = 1.49). Therefore, H3 was partially supported. This means that for every unit change in X (perceived usability), there is a corresponding change in Y (time). That is, for every unit increase in usability for the egocentric view, participants were nearly 68 s faster. In contrast, there was no significant relationship between usability in the exocentric view and time performance.

## 5. Discussion

Conceptually, as an interactive technique, the exocentric view had several advantages over the egocentric view. Therefore, it was anticipated that the exocentric view would enable superior task performance, resulting in faster task completion time (efficiency) and higher usability overall (H1 and H2). These expectations were supported in the present study.

This study used 3D VR simulation to assess whether the visualisation and immersive analytics for biomedical and genomics data would be consistent with the previous literature. This study measured usability by looking at the time performance and user preference, including the extent to which the exocentric view can facilitate easy and efficient data interpretation. Average ease-of-use scores confirmed that participants performed significantly faster in the exocentric view than in the egocentric view. They also rated higher on the exocentric view because they interacted with the visualisation more smoothly with the exocentric view.

Extracting health data information using immersive visualisation may require a trade-off between usability and speed, i.e., some features may require users to take longer to complete tasks [11] This could explain the patterns of results observed in the present study, with the egocentric view displaying a longer time to complete the visual analytics task. In other words, participants performed better and were more efficient in the visual analytics task when they used the exocentric view. These findings supported the previous study, which illustrated that users were required to devote more time to the egocentric view due to a significant perception distortion, resulting in the extra effort of body interactions, such as users having to turn their heads more frequently [12]. Another possible explanation is that it was due to the large-scale visualisation, which maximised the size of the distances relative to the participant’s field of view at the default viewing distance. While it is feasible for participants to move closer to the visualisations to obtain a more precise scale, doing so may be inconvenient or time-consuming. On the other hand, the exocentric view presented health data without distortion, resulting in faster time performance for participants. It is also crucial to note that this could result from a carryover effect. Contrary to the findings in [13], the exocentric view was more effective than the egocentric view in working with visual tasks in a virtual environment.

Average ease-of-use scores revealed that participants preferred interacting with the exocentric view over the egocentric view, which was in line with expectations. Results from the data analysis showed that the participants highly regard the potential ease of usability of the exocentric view of the VROOM tool for distinguishing patient-to-patient comparisons based on the modelling of their genetic and biomedical information. Qualitative comments also highlighted participants’ preference for the exocentric view over the egocentric view. For example, a few participants noted that the exocentric view was more natural, accessible, and easier to navigate for health data displayed in front of them, rather than having to look around. In comparison, the egocentric technique was noted to be disorienting and significantly more difficult to locate data and follow instructions. Another reason participants preferred the exocentric view was because the egocentric view causes them to feel more motion sickness due to constantly needing to turn their heads around and behind to complete tasks in the heavy VR head-mounted device.

The findings indicated that the exocentric view is the preferred option and more efficient for health data analysis than the egocentric view. A well-structured and visible exocentric view enabled all data items to be organised and displayed in the users’ field of vision, allowing them to interpret data in a straightforward and meaningful manner. The simplicity of visualising data in the exocentric view on the VROOM tool [8] has facilitated users to complete tasks promptly and with complete concentration.

## 6. Conclusions

Although VR has sparked considerable interest in the medical field, few usability studies have been conducted to evaluate such visualisations. This paper presents a study on the exocentric and egocentric views in the VR environment, where we focus on genomics and biomedical data immersive analytics. Our study indicates superior performance and ease-of-use scores in the exocentric view compared to the egocentric view. The results of our study also support the hypothesis that the usability of the egocentric view is positively related to the time performance of the visual analytics tasks. We believe that the outcome of this study will provide a solid guideline for the design of visualisation in the VR environment.

## Figures and Tables

**Table 1:** Visual analytics tasks and items.

*Tasks*
Task 1: Press the TOOLS button. Then, open the Patient to Patient button.
Task 2: Select an intermediate-risk male patient between the ages of 50 and 70 by using your index finger and drag them into the left panel.
Task 3: Select another intermediate-risk male patient between the ages of 50 and 70 by using your index finger and drag them into the right panel.
*Question*: What is the R2 value on the scatterplot?
Task 4: Select a high-risk female patient between the ages of 40 and 60 by using your index finger and drag them into the left panel.
Task 5: Select another high-risk female patient between the ages of 40 and 60 by using your index finger and drag them into the right panel.
*Question*: What is the R2 value on the scatterplot?
*Perceived ease of use (adapted from* [29]*)*
I found the egocentric 3D web-based VR simulation awkward to use.
Learning to interact with the exocentric 3D web-based VR simulation was easy for me.
Interacting with the exocentric 3D web-based VR simulation is often frustrating.
I found it easy to get the exocentric 3D web-based VR simulation to do what I wanted it to do.
Overall, the exocentric 3D web-based VR simulation was easy to use.

**Table 2:** Paired samples test for time performance of exocentric and egocentric views.

		Paired Differences					t	df	Significance	
		Mean	Std. Deviation	Std. Error Mean	95% Confidence Interval of the Difference				One-Sided *p*	Two-Sided *p*
					Lower	Upper				
Pair 1	Egocentric Time in Secs—Exocentric Time in Secs	113.76895	145.56483	23.61373	65.92298	161.61491	4.818	37	<0.001	<0.001

**Table 3:** Paired samples test for ease-of-use scores of exocentric and egocentric views.

		Paired Differences					t	df	Significance	
		Mean	Std. Deviation	Std. Error Mean	95% Confidence Interval of the Difference				One-Sided *p*	Two-Sided *p*
					Lower	Upper				
Pair 1	Q90 Average—Q87 Average	0.3947	0.8677	0.1408	0.1095	0.6800	2.804	37	0.004	0.008

**Table 4:** Regression analysis for the ease-of-use scores for exocentric view and egocentric view.

ANOVA ^a^					
Model	Sum of Squares	df	Mean Square	F	Sig.
Regression	209,037.243	2	104,518.621	6.362	0.004 ^b^
Residual	574,960.134	35	16,427.432		
Total	783,997.377	37			

^a^ Dependent Variable: Egocentric Minus Exocentric, ^b^ Predictors: (Constant), Q90 Average, Q87 Average.

**Table 5:** Coefficients analysis.

Coefficients ^a^							
	Unstandardized Coefficients		Standardized Coefficients			95.0% Confidence Interval for B	
Model	B	Std. Error	Beta	t	Sig.	Lower Bound	Upper Bound
Constant	398.772	108.609		3.672	<0.001	178.284	619.260
Q87 Average	−67.821	24.956	−0.480	−2.718	0.010	−118.484	−17.159
Q90 Average	−10.952	32.529	−0.059	−0.337	0.738	−76.988	55.085

^a^ Dependent Variable: Egocentric Minus Exocentric.

## Data Availability

Publicly available datasets were analysed in this study. This data can be found here: https://www.cancer.gov/ccg/research/genome-sequencing.
